# Predictors of Lymph Node Involvement by Soft Tissue Sarcoma of the Trunk and Extremity: An Analysis of the National Cancer Database

**DOI:** 10.7759/cureus.6038

**Published:** 2019-10-30

**Authors:** Joseph A Miccio, Vikram Jairam, Sarah Gao, Alexander Augustyn, Oluwadamilola T Oladeru, Benjamin E Onderdonk, Mudit Chowdhary, Dale Han, Sajid Khan, Gary Friedlaender, Dieter M Lindskog, Hari A Desphande, Heather Osborn, Kenneth B Roberts, Kirtesh R Patel

**Affiliations:** 1 Department of Therapeutic Radiology, Yale School of Medicine, New Haven, USA; 2 Department of Radiation Oncology, The University of Texas MD Anderson Cancer Center, Houston, USA; 3 Department of Radiation Oncology, Massachusetts General Hospital/Harvard Medical School, Boston, USA; 4 Department of Radiation and Cellular Oncology, University of Chicago Medical Center, Chicago, USA; 5 Department of Radiation Oncology, Rush University Medical Center, Chicago, USA; 6 Department of Surgical Oncology, Oregon Health and Science University, Portland, USA; 7 Department of Surgery, Yale School of Medicine, New Haven, USA; 8 Department of Orthopaedics and Rehabilitation, Yale School of Medicine, New Haven, USA; 9 Department of Medical Oncology, Yale School of Medicine, New Haven, USA; 10 Department of Otolaryngology, Yale School of Medicine, New Haven, USA; 11 Department of Radiation Oncology, Yale School of Medicine, New Haven, USA

**Keywords:** soft tissue sarcoma, trunk, extremity, lymph node metastasis, national cancer database (ncdb)

## Abstract

Background and Objectives

Lymph node metastases (LNM) in soft tissue sarcoma (STS) of the trunk and extremity are rare but are associated with worse survival. Established risk factors for LNM in this group are based on small institutional retrospective reviews. This study identifies the risk factors associated with LNM in otherwise non-metastatic trunk/extremity STS patients using the National Cancer Database (NCDB) and sought out to delineate a high-risk group that may be considered for pathologic nodal evaluation.

Methods

The files of 10,731 patients with STS of the trunk/extremity without distant metastasis from 2004 - 2015 were evaluated. Exclusion criteria included neoadjuvant therapy and a lack of pathologic nodal evaluation. Univariate and multivariable logistic regression models were performed to evaluate variables associated with LNM.

Results

Of the total of 10,731 patients, 223 (2.1%) had LNM. On multivariable analysis, LNM was associated with Grade 3 tumors (odds ratio (OR) 15.6, 95% confidence interval (CI) 6.36 - 38.04, p < 0.001) and clear cell/angiosarcoma/rhabdomyosarcoma/epithelioid (CARE) histology (OR 4.72, 95% CI 3.35 - 6.66, p < 0.001), lymphovascular invasion (LVI) (OR 5.86, 95% CI 3.33 - 10.31, p < 0.001, and bone invasion (BI) (OR 2.73, 95% CI 1.32 - 5.61, p = 0.006). Patients with Grade 3 CARE tumors (n = 402) had an 11.9% risk of LNM vs. 1.7% of adults without all these characteristics (p < 0.001). Patients with Grade 3 CARE tumors and either LVI or BI (n = 36) had a 33.3% risk of LNM.

Conclusions

High-grade and CARE histology are associated with LNM in STS. Adult patients with both features have an overall 11.9% risk of LNM and may be considered for pathologic LN assessment, particularly with the presence of LVI or BI.

## Introduction

Lymph node metastases (LNM) in adults with trunk/extremity soft tissue sarcoma (STS) are rare, generally affecting < 5% of STS patients [1-3]. However, patients with lymph node involvement have worse overall survival. Several retrospective studies have shown that patients with LNM metastasis have poorer survival [3-4], and the American Joint Commission on Cancer (AJCC) has changed the group staging for lymph node-positive patients with trunk/extremity sarcoma from Stage III to Stage IV. Furthermore, retrospective evidence suggests that lymphadenectomy in patients with LNM from STS is necessary for long-term survival [1-3, 5-7]. Thus, it is important to identify patients with STS at high risk for LNM, as these patients may be candidates for pathologic lymph node assessment.

Previous studies defining risk factors for nodal metastasis are primarily based on small, single-institution retrospective studies [1, 3, 7-8]. These series have reported an incidence of lymph node metastasis of 2% - 7%. Reports have also identified several variables associated with the development of lymph node metastasis, including histologic subtype, tumor grade [1, 8-9], patient age [2, 8], and primary tumor size [7-8].

The aim of this study is to identify risk factors associated with lymph node metastasis in STS patients without distant metastasis using the National Cancer Database (NCDB). We also aimed to identify a clinically relevant high-risk group with an increased risk of harboring disease in regional lymph nodes that may benefit from pathologic LN (lymph node) assessment.

## Materials and methods

The NCDB is a joint project of the Commission on Cancer (CoC) of the American College of Surgeons and the American Cancer Society, which consists of de-identified information regarding patient demographics, tumor characteristics, first-course treatment for the corresponding diagnosis, and survival for approximately 70% of the United States (US) population [10]. All pertinent cases are reported regularly from CoC-accredited centers and compiled into a unified dataset, which is then validated. The NCDB contains information not included in the Surveillance, Epidemiology, and End Results (SEER) database, including details regarding the use of systemic therapy. The data used in the study were derived from a de-identified NCDB file (2004 - 2015). The American College of Surgeons and the CoC have not verified and are neither responsible for the analytic or statistical methodology employed, nor the conclusions drawn from these data by the investigators. As all patient information in the NCDB database is de-identified, this study was exempt from institutional review board evaluation.

**Figure 1:**
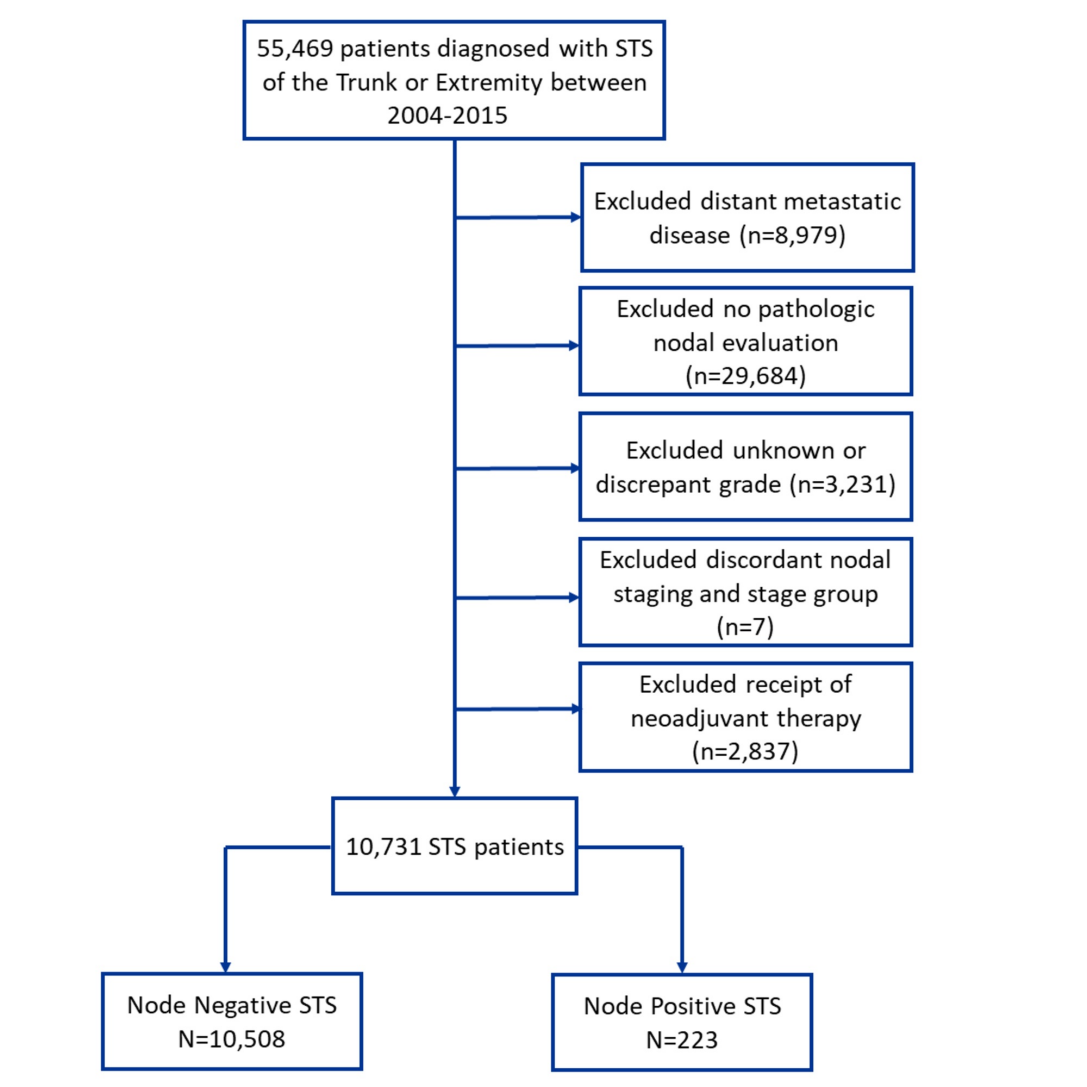
Consolidated standards of reporting trials diagram delineating cohort selection The final cohort consisted of 10,508 trunk/extremity soft tissue sarcoma patients without lymph node metastasis (97.9%) and 223 with lymph node metastasis (2.1%). STS: soft tissue sarcoma

The 2015 NCDB Participant User File contains 96,522 patients with soft tissue sarcoma diagnosed between 2004 and 2015. We included only patients with primary site codes of C471, C472, C476, C478, C479, C491, C492, C496, C498, and C499, corresponding to the ICD-O-3 codes included for trunk/extremity STS via the American Joint Committee on Cancer (AJCC), 8th edition (n = 55,469) [11]. Other exclusion criteria included distant metastatic disease (n = 8,979), lack of pathologic nodal evaluation (n = 29.684), unknown or discrepant grade (n = 3,231), discordant nodal status and stage group (n = 7), and any patients receiving neoadjuvant therapy or if receipt of neoadjuvant therapy was unknown (n = 2,837). The remaining patients were categorized as either having a pathologically node-positive or node-negative status. Figure 1 shows the Consolidated Standards of Reporting Trials (CONSORT) diagram for the study [12]. Our final cohort consisted of 10,731 patients, 223 of whom had pathologic lymph node involvement.

The following disease-related variables were evaluated for association with LN involvement: grade, histology, tumor size, tumor location (trunk vs. extremity), LVI, BI, and neurovascular invasion (NVI). We also included the demographic factors of age, sex, and race. Age was evaluated both as a continuous variable and categorical variable (< 18 and ≥ 18). The histologies of clear cell, angiosarcoma, rhabdomyosarcoma, and epithelioid (CARE) were grouped as “CARE” histologies, given their higher propensity for LNM as previously described [4, 13] Other categories for histology included synovial sarcoma, given conflicting reports regarding its association with LNM [14-16], and “other.” Univariate comparisons between the LNM cohort and node-negative cohort were made via the Chi-square test. Univariate and logistic regression models were performed to evaluate variables associated with pathologic node positivity. Variables associated with LNM with a P-value < 0.1 were included in the multivariable logistic regression model using a forward stepwise method, as were variables that were previously shown to have an effect on LNM, such as tumor size [7-8]. Odds ratios and 95% confidence intervals were generated. P-values were derived from two-tailed tests. The analysis was performed using STATA, version 13 (StataCorp LLC, College Station, TX) and P-values < 0.05 were considered statistically significant.

## Results

Ten thousand seven hundred and thirty-one soft tissue sarcoma patients meeting the inclusion criteria were identified which included 10,508 patients without LNM (97.9%) and 223 patients with LNM (2.1%). The cohort characteristics are detailed in Table 1. The LNM cohort had a higher proportion of pediatric cases (16, 7.2%) compared with the node-negative group (235 cases, 2.2%, p < 0.001), but race was balanced between the node-positive and node-negative cohorts (p = 0.449). The LNM cohort, compared to the node-negative group, had a higher proportion of primary tumors that were in the trunk (47 cases, 21.1% vs. 1,433 cases, 13.6%, p < 0.001), Grade 3 (198 cases, 88.8% vs. 5,929 cases, 56.4%, p < 0.001), CARE histology (56 cases, 25.1% vs 458 cases, 4.4%, p < 0.001), LVI (26 cases, 11.7% vs. 199 cases, 1.9%, p < 0.001), NVI (7 cases, 3.1% vs. 113 cases, 1.1%, p = 0.008), and BI (10 cases, 4.5% vs. 105 cases, 1.0%, p < 0.001). The CARE histology category contained 61 (11.9%) clear cell cases, 180 (35.0%) angiosarcoma cases, 139 (27.0%) rhabdomyosarcoma cases, and 134 (26.1%) epithelioid sarcoma cases, and there were 446 cases of synovial sarcoma.

**Table 1:** Cohort Characteristics for Trunk/Extremity Soft Tissue Sarcoma Patients With or Without Lymph Node Metastasis

Variable	Node-negative	%	Node-positive	%	P-value
Total	10,508	97.9	223	2.1	
Age (years)					< 0.001
< 18	235	2.2	16	7.2	
≥ 18	10,237	97.8	207	92.8	
Sex					0.999
Male	5,654	53.8	120	53.8	
Female	4,854	46.2	103	46.2	
Race					0.449
White	8,743	83.2	183	82.1	
Black	1,192	11.3	20	13.5	
Asian	283	2.7	7	3.1	
Unknown	290	2.8	3	1.4	
Location					0.001
Trunk	1,433	13.6	47	21.1	
Extremity	9,076	86.4	176	78.9	
Size					< 0.001
≤ 5 cm	9.926	94.5	195	87.4	
> 5 cm	52	0.5	4	1.8	
Unknown	530	5.0	24	10.8	
Grade					< 0.001
I	2,850	27.1	5	2.2	
II	1,729	16.5	20	9.0	
III	5,929	56.4	198	88.8	
Histology					< 0.001
Clear cell, angiosarcoma, rhabdomyosarcoma, epithelioid (CARE) histology	458	4.4	56	25.1	
Synovial	436	4.1	10	4.5	
Other	9,614	91.5	157	70.4	
Lymphovascular Invasion					< 0.001
Absent	2,938	28.0	38	17.0	
Present	199	1.9	26	11.7	
Unknown	7,371	70.1	159	71.3	
Neurovascular Invasion					0.008
Absent	3,274	31.2	60	26.9	
Present	113	1.1	7	3.1	
Unknown	7,121	67.8	156	70.0	
Bone Invasion					< 0.001
Absent	4,722	44.9	117	54.5	
Present	105	1.0	10	4.5	
Unknown	5,681	54.1	96	43.1	

On univariate analysis (Table 2), older patients and patients with extremity tumors were less likely to have nodal metastasis from STS (OR 0.99, 95% CI 0.986 - 0.999, p = 0.043 and OR 0.59, 95% CI 0.43 - 0.82, p = 0.002, respectively). Race and sex were not associated with nodal positivity (p > 0.1). High grade (Grade 2 vs. Grade 1, OR 6.59, 95% CI 2.47 - 17.60, p < 0.001 and Grade 3 vs. Grade 1, OR 19.03, 95% CI 7.83 - 46.29, p < 0.001), LVI (OR 10.10, 95% CI 6.01 - 16.98, p < 0.001), NVI (OR 3.38, 95% CI 1.51 - 7.56, p = 0.003), BI (OR 3.84, 95% CI 1.96 - 7.54, p < 0.001), and tumor size > 5 cm (OR 3.92, 95% CI 1.40 - 10.93, p = 0.009) were all associated with LNM. CARE histology was also associated with LNM (CARE histology vs. other, OR 7.49, 95% CI 5.44 - 10.3, p < 0.001), but the synovial sarcoma histology was not (synovial sarcoma vs. other, OR 1.4, 95% CI 0.74 - 2.68, p = 0.666).

**Table 2:** Univariate and Multivariable Logistic Regression Predicting for Lymph Node Metastasis CARE: clear cell, angiosarcoma, rhabdomyosarcoma, and epithelioid; CI: confidence interval

Variable	Univariate Odds Ratio (95% CI)	P-value	Multivariable Odds Ratio (95% CI)	P-value
Age (Continuous)	0.993 (0.986 – 0.999)	0.043	0.99 (0.987 – 1.001)	0.087
Sex			NA	
Male	Reference			
Female	1.00 (0.77 – 1.30)	0.999		
Race			NA	
White	Reference			
Black	1.20 (0.81 – 1.78)	0.355		
Asian	1.18 (0.55 – 2.54)	0.668		
Unknown	0.49 (0.16 – 1.56)	0.228		
Location				
Trunk	Reference		Reference	
Extremity	0.59 (0.43 – 0.82)	0.002	0.70 (0.50 – 0.99)	0.042
Size				
≤ 5 cm	Reference		Reference	
> 5 cm	3.92 (1.40 – 10.93)	0.009	4.12 (1.42 – 11.99)	0.009
Unknown	2.31 (1.50 – 3.55)	< 0.001	2.41 (1.52 – 3.80)	< 0.001
Grade				
I	Reference		Reference	
II	6.59 (2.47 – 17.60)	< 0.001	6.05 (2.26 – 16.23)	< 0.001
III	19.03 (7.83 – 46.29)	< 0.001	15.55 (6.36 – 38.04)	< 0.001
Histology				
Other	Reference		Reference	
CARE	7.49 (5.44 – 10.30)	<0.001	4.72 (3.35 – 6.66)	< 0.001
Synovial	1.40 (0.74 – 2.68)	0.303	0.98 (0.50 – 1.93)	0.953
Lymphovascular Invasion				
Absent	Reference		Reference	
Present	10.10 (6.01 – 16.98)	< 0.001	5.86 (3.33 – 10.31)	< 0.001
Unknown	1.67 (1.17 – 2.38)	0.005	2.17 (1.43 – 3.29)	< 0.001
Neurovascular Invasion				
Absent	Reference		Reference	
Present	3.38 (1.51 – 7.56)	0.003	1.16 (0.47 – 2.83)	0.751
Unknown	1.20 (0.89 – 1.61)	0.245	1.71 (1.17 – 2.51)	0.006
Bone Invasion				
Absent	Reference		Reference	
Present	3.84 (1.96 – 7.54)	< 0.001	2.73 (1.32 – 5.61)	0.006
Unknown	0.68 (0.52 – 0.90)	0.006	0.40 (0.28 – 0.58)	< 0.001

Table 2 also shows the multivariable logistic regression analysis. On multivariable analysis, several variables remained associated with LNM. These included Grade 3 tumors (Grade 3 vs. Grade 1, OR 15.55, 95% CI 6.36 - 38.04, p < 0.001), LVI (OR 5.86, 95% CI 3.33 - 10.31, p < 0.001,), BI (OR 2.73, 95% CI 1.32 - 5.61, p = 0.006), and tumor size > 5 cm (OR 4.12, 95% CI 1.42 - 11.99, p = 0.009). CARE histology was also strongly associated with LNM (OR 4.72, 95% CI 3.35 - 6.66, p < 0.001), while synovial sarcoma was not (OR 0.98, 95% CI 0.50 - 1.93, p = 0.953), and NVI was not associated with LNM on multivariable analysis (p > 0.1).

**Figure 2:**
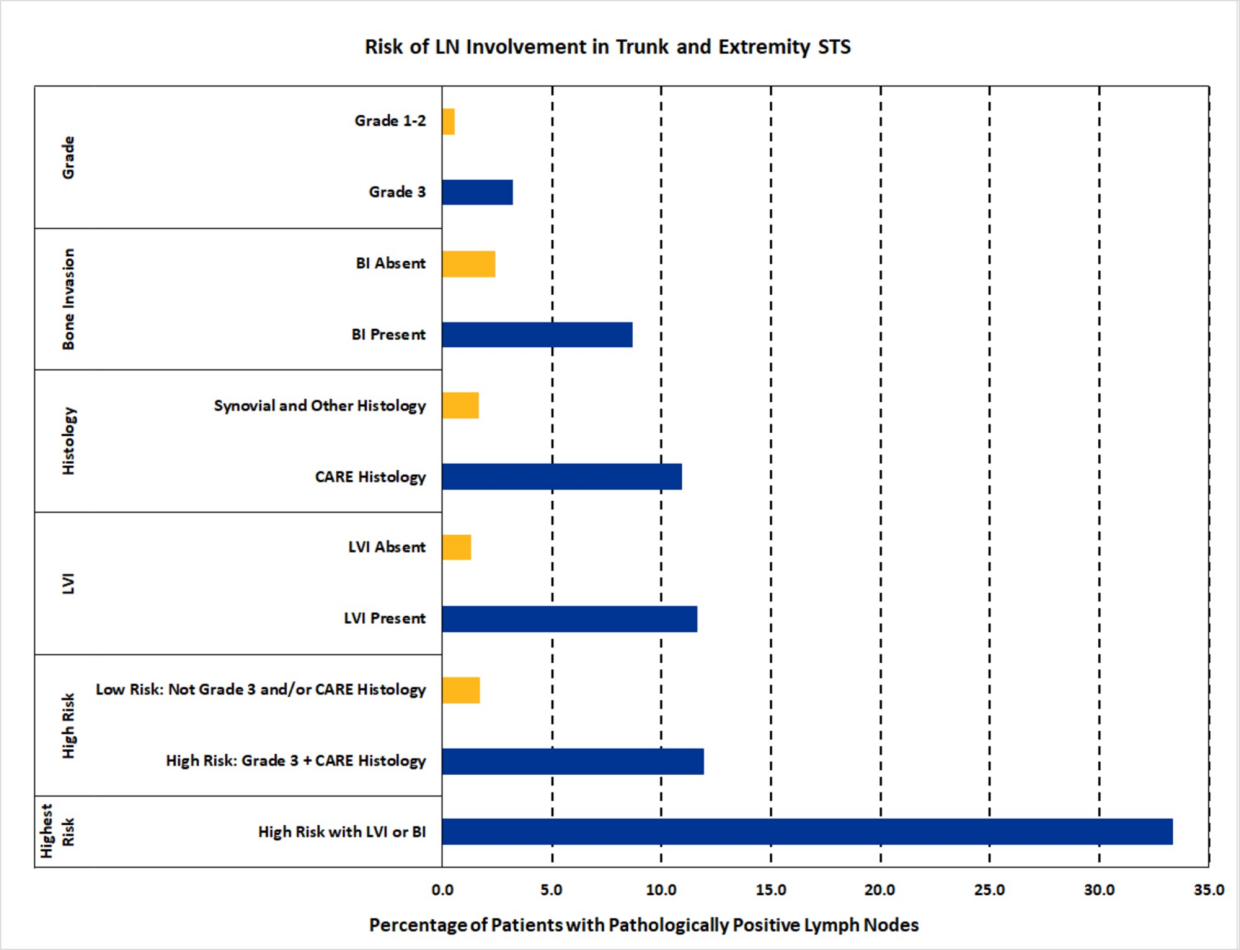
Risk of Lymph Node Involvement in Trunk and Extremity Soft Tissue Sarcoma (STS) CARE: clear cell, angiosarcoma, rhabdomyosarcoma, and epithelioid sarcoma; LN: lymph node; LVI: lymphovascular invasion

Adult patients with Grade 3 tumors and CARE histology were identified as a “high-risk” subset. A total of 402 patients in our cohort were designated as high-risk, and this subset of patients had a greater risk of LNM relative to adult patients without both factors: 11.9% vs. 1.7%, p < 0.001. Furthermore, high-risk patients with either LVI or BI exhibited an extremely high rate of LNM compared to high-risk patients without LVI or BI (33.3% vs. 9.8%, p < 0.001) (Figure 2).

## Discussion

This is the first large scale study of clinical and histologic predictors of LNM in trunk/extremity STS. LN involvement in non-metastatic STS is associated with high-grade, CARE histology, LVI, and BI. High-risk patients - defined as high-grade with CARE histology - have an 11.9% risk of LN involvement and can be considered for pathologic LN assessment, particularly if they exhibit LVI or BI, which increased LNM rates to 33%. Identifying positive lymph nodes in STS may inform prognosis and would likely influence patient management, given the retrospective evidence suggesting a survival benefit for patients with LNM receiving regional lymphadenectomy [1-3, 5-6].

The previous studies of LNM in STS primarily evaluated histology and grade as risk factors for nodal involvement [1, 3, 7, 16-19]. To our knowledge, the first study of LNM in STS was published in 1978, where a review of over 3,000 cases of STS identified rhabdomyosarcoma and synovial sarcoma to be associated with increased risk of LNM [16]. In 1987, a cohort study of 323 patients showed that high-grade, rhabdomyosarcoma histology, and epithelioid histology were associated with LNM [7]. A larger study of 1,772 patients showed that angiosarcoma, rhabdomyosarcoma, and epithelioid sarcoma had higher rates of LNM [3]. Another study of 1,066 patients confirmed the previously established risk factors and also showed that clear cell sarcoma increased LNM [1].

A more recent publication by Keung et al. using the NCDB showed that lymph node positivity had an impact on survival for non-metastatic patients with certain histologic subtypes of sarcoma [4]. This suggests that pathologic confirmation of nodal disease has a prognostic value, and several retrospective series have shown regional lymphadenectomy to improve survival [1-3, 5, 6]. However, there are no prospective data that show regional lymphadenectomy improves outcomes for patients undergoing surgery for STS. Given the rarity of these tumors and the inherent difficulty of conducting prospective studies, the National Comprehensive Cancer Network (NCCN) has recommended lymphadenectomy for patients with nodal disease based on these retrospective studies. Thus, identifying patients at high-risk for harboring nodal disease is imperative.

This study adds to the current literature by confirming previously identified high-risk histologies and identifying new risk factors for LNM in STS of the trunk/extremity (BI). Furthermore, this study identifies a high-risk cohort with an 11.9% risk of LNM based on factors that can be determined preoperatively with biopsy alone. This high-risk cohort of patients, particularly those with LVI or BI, may benefit from sentinel node biopsy, and, if positive, should be considered for regional lymphadenectomy based on current NCCN recommendations derived from the aforementioned retrospective studies. Of the 223 pathologically node-positive patients in our cohort, only 114 patients were coded as clinically node-positive, 47 patients were coded as clinically node-negative, and the remainder did not have a clinical nodal stage recorded. This suggests that at least 21.1% of the patients (47/223) with nodal disease had no clinical evidence of nodal involvement. It is likely that in some of these cases, nodal metastases were discovered incidentally during resection of a primary tumor, which has been discussed in prior series [2, 4], but it is possible that some of these patients had a sentinel lymph node biopsy (SLNB). Although the role of SLNB in STS has not yet been determined, a prospective study showed that six of 12 patients with clear cell sarcoma had clinically occult LNM identified by SLNB [20], which suggests the technique may be useful in patients at high risk for LNM.

A few limitations should be discussed. As is the case in any retrospective review, we may not have been able to control for unknown variables that may have been unbalanced between the cohorts. Furthermore, many of the variables examined had unknown/missing values, including 5.2% of tumor size, 70.2% of LVI, 67.8% of NVI, and 53.8% of BI. We excluded patients who received neoadjuvant therapy to avoid treatment effect on LNM; however, this greatly reduced the number of patients included in the study, including patients likely with larger tumors, given only 56 patients in our cohort had a primary tumor > 5 cm. Furthermore, the NCDB does not record data for lymph node size, the presence of extracapsular extension, or the type of procedure conducted for lymph node evaluation. We did not evaluate survival in our cohort since we excluded patients receiving neoadjuvant treatment, which is a group with worse prognostic factors and possibly worse overall survival (OS) [21]. Despite these limitations, this is the largest study of risk factors for LNM in trunk/extremity STS, and the factors identified in this study can be used by practitioners to select patients at high risk for harboring LNM who may benefit from SLNB, even in the absence of clinical evidence of nodal involvement.

6.3.6

## Conclusions

This NCDB study of non-metastatic STS of the trunk and extremity shows that high-grade and CARE histology were associated with pathologically-proven LNM. Adult patients with both features have an overall 11.9% risk of LNM and may be considered for pathologic LN assessment, particularly with the presence of LVI or BI, where the risk of LNM is > 30%. Patients with proven LNM in the absence of distant metastatic disease can be considered for regional lymphadenectomy based on current NCCN guidelines.
